# Electrophysiological brain signatures for the classification of subjective cognitive decline: towards an individual detection in the preclinical stages of dementia

**DOI:** 10.1186/s13195-019-0502-3

**Published:** 2019-06-01

**Authors:** David López-Sanz, Ricardo Bruña, María Luisa Delgado-Losada, Ramón López-Higes, Alberto Marcos-Dolado, Fernando Maestú, Stefan Walter

**Affiliations:** 1 Laboratory of Cognitive and Computational Neuroscience (UCM-UPM), Centre for Biomedical Technology (CTB), Technical University of Madrid (UPM), Madrid, Spain; 2 Department of Experimental Psychology, Complutense University of Madrid (UCM), Madrid, Spain; 3 CIBER-BBN: Networking Research Center on Bioengineering, Biomaterials and Nanomedicine, Zaragoza, Spain; 4 Neurology Department, Clinico San Carlos Hospital, Madrid, Spain; 5 Department of Epidemiology and Biostatistics, University of California San Francisco, San Francisco, USA; 6 Dept. of Preventive Medicine and Public Health, University Rey Juan Carlos, Madrid, Spain

**Keywords:** Neuroimaging, Magnetoencephalography, Alpha band, Subjective cognitive decline, Alzheimer’s disease

## Abstract

**Background:**

Alzheimer’s disease (AD) prevalence is rapidly growing as worldwide populations grow older. Available treatments have failed to slow down disease progression, thus increasing research focus towards early or preclinical stages of the disease. Subjective cognitive decline (SCD) is known to increase the risk of developing AD and several other negative outcomes. However, it is still very scarcely characterized and there is no neurophysiological study devoted to its individual classification which could improve targeted sample recruitment for clinical trials.

**Methods:**

Two hundred fifty-two older adults (70 healthy controls, 91 SCD, and 91 MCI) underwent a magnetoencephalography scan. Alpha relative power in the source space was employed to train a LASSO classifier and applied to distinguish between healthy controls and SCD. Moreover, MCI participants were used to further validate the previously trained algorithm.

**Results:**

The classifier was significantly associated to SCD with an AUC of 0.81 in the whole sample. After randomly splitting the sample in 2/3 for discovery and 1/3 for validation, the newly trained classifier was also able to correctly classify SCD individuals with an AUC of 0.75 in the validation sample. The regions selected by the algorithm included medial frontal, temporal, and occipital areas. The algorithm trained to select SCD individuals was also significantly associated to MCI diagnostic.

**Conclusions:**

According to our results, magnetoencephalography could be a useful tool for distinguishing individuals with SCD and healthy older adults without cognitive concerns. Furthermore, our classifier showed good external validity, being not only successful for an unseen SCD sample, but also in a different population with MCI cases. This supports its utility in the context of preclinical dementia. These findings highlight the potential applications of electrophysiological techniques to improve sample recruitment at the individual level in the context of clinical trials.

**Electronic supplementary material:**

The online version of this article (10.1186/s13195-019-0502-3) contains supplementary material, which is available to authorized users.

## Introduction

Modern societies are experiencing a dramatic increase in life expectancy, even in lower income countries the proportion of people aged over 60 years will increase during the next decades, which together with decreased birth rates is leading to an unprecedented population aging. As a consequence, the prevalence of diseased population and the medical costs associated to their treatment are rapidly increasing [1, 2]. This context has raised numerous concerns about the sustainability of current clinical practice, mainly focused on expensive and chronic healthcare of fully developed syndromes. Consequently, research efforts are shifting towards early detection [3], which could reduce the cost associated to disease treatment by promoting and improving the efficacy of targeted early intervention programs. More concretely, Alzheimer’s disease (AD) dementia will be one of the main challenges for health systems during the decades to come, since its prevalence is expected to nearly triple by 2050 [4]. Several clinical trials and drugs available have consistently failed to achieve a significant interruption or slowing of the disease pathology or clinical symptoms progression [5], which is increasing the interest in its preclinical stages. However, our ability to distinguish at-risk individuals in the earliest stages of the disease is still relatively limited. Some at-risk conditions have been described at the individual level, yet reliable markers to classify subjects on an individual basis remain still relatively unknown.

There is accumulating evidence that subjective cognitive decline (SCD) might be one of the earliest indicators of the pathological cascade underlying dementia [6]. SCD is characterized by a subjective feeling of cognitive worsening in comparison to a previous state, in the absence of objective neuropsychological deficits. Therefore, SCD elders are at an increased conversion risk to mild cognitive impairment (MCI) and dementia varying between a 2-fold and a 5-fold increase depending on the study [5–8]. Furthermore, SCD has been related not only to dementia [9], but also to other relevant age-related outcomes such as increased depression risk [10], impaired higher level functional capacity [11] and even increased vulnerability as reflected by higher risk of nursing home placement [12]. Furthermore, despite some inconsistent results [13], SCD has also been found to be predictive of increased all-cause mortality in the older adult population [14]. Altogether, these findings highlight the relevance of identifying SCD as a potential early marker of forthcoming decline.

Neuroscience has only recently started to explore whether differences in brain functioning or structure can be identified at the group level in SCD population. Interestingly, there is increasing evidence showing that elders with SCD, as a group, show brain patterns resembling of those exhibited by AD patients, such as reduced metabolism [15] or gray matter atrophy over medial temporal structures [16]. Electrophysiological studies have consistently proven the relevance of power spectrum alterations in the AD continuum, showing abnormal relative power distribution affecting both slow and fast brain rhythms [17]; furthermore, similar alterations have also been found in MCI patients [18] and SCD [19]. Despite the fact that several alterations have been identified at the group level in SCD individuals (CSF, amyloid-PET deposition, power alterations, etc.), to the best of our knowledge, it has not been studied whether these differences translate into individual classification in independent samples. Detection of SCD by objective means could potentially represent an initial step for targeted subject selection in the context of clinical trials, specific interventions in the early stages, and individual diagnosis. [20]. In this vein, magnetoencephalography (MEG) is not yet established as a biomarker for AD diagnosis, although recent advances in the study of AD using this technique [21]. All the above-mentioned pathophysiological alterations have implications in synaptic transmission at a very early stage of the disease. MEG is able to measure tiny variations in the magnetic fields arising from neuronal post-synaptic activity localized over cortical sources mainly; henceforth, it is sensitive to subtle alterations in this activity with extremely high temporal precision and good spatial resolution. This makes MEG a suitable technique for a potential biomarker identification in the context of dementia.

To this aim, we tried to train a classifier able to discriminate healthy elders with and without SCD in independent samples using their brain power spectral properties and basic demographic information such as age and gender. Furthermore, we validated our classifier in another completely unseen sample of amnestic-MCI participants under the assumption that the previously trained algorithm should also be able to significantly differentiate later stages of the progression to dementia. More concretely, source-space alpha band power was selected as our main feature as it has been shown to be specifically affected in SCD by two different studies, by our group and others that discarded alterations in other frequency ranges [19, 22].

## Material and methods

### Participants

A total of 161 community-dwelling elders (mean age 71.6, SD of 4.9 years) recruited in three centers in Madrid (Spain) enrolled in this study after signing an informed consent. The study protocol was approved by the Hospital Universitario San Carlos ethics committee. In all the procedures, participants were divided into two groups, healthy control group without cognitive concerns (HC, *n* = 70) and older adults with subjective cognitive decline (SCD, *n* = 91). All subjects underwent a neuropsychological assessment to confirm their normal cognitive state, an MRI scan and lastly an MEG recording. The exclusion criteria included the following: (1) history of psychiatric (e.g., depression), or neurological disorders, or drug consumption that could affect MEG activity such as cholinesterase inhibitors; (2) evidence of infection, infarction, or focal lesions in a T2-weighted scan within 2 months before MEG acquisition; (3) a modified Hachinski score equal to 5 or higher; (4) alcoholism, chronic use of anxiolytics, neuroleptics, narcotics, anticonvulsants, or sedative hypnotics. Furthermore, additional analysis to rule out other possible causes of cognitive decline such as B12 vitamin deficit, diabetes mellitus, thyroid problems, syphilis, or human immunodeficiency virus (HIV) was conducted. Furthermore, 91 additional participants (mean age 72.8, SD of 4 years) with amnestic-MCI and recruited using the same exact criteria were included in the study as an external validation sample.

### Clinical assessment

All the subjects underwent an initial screening to assess their overall state including The Mini Mental State Examination (MMSE), Functional Assessment Questionnaire (FAQ), the Hachinski Ischemic Scale, and the Geriatric Depression Scale-Short Form as a screening to ensure normal functioning level and preserved vascular health. After the initial screening, each participant completed a neuropsychological assessment including evaluation of their memory, executive functions, language, and praxis.

All participants in the HC and SCD groups had normal cognitive performance in the standardized assessment with respect to their age and education. Expert clinicians gathered information about cognitive concerns for each participant during an interview, in which subjects self-reported whether they felt a significant cognitive decline with respect to their previous performance level. During the interview, the SCD-questionnaire [23] was applied to each participant, and although diagnostic was not only based on the questionnaire score, it was considered by clinicians for the evaluation of SCD presence. The final group assignment was made according to a multidisciplinary consensus by neuropsychologists, psychiatrists, and neurologists. Several possible confounders of SCD such as medication, psycho-affective disorders, or relevant medical conditions were taken into account for the decision. According to recent criteria proposed for studying SCD, all the participants were older than 60 at onset of SCD, which occurred within the last 5 years [6].

For the validation sample, MCI diagnosis was established according to the criteria proposed by Petersen [24].

### MRI acquisition

Each subject completed an MRI scan in order to use the images for the source reconstruction analysis. T1-weighted images were acquired in a General Electric 1.5 Tesla magnetic resonance, using a high-resolution antenna and a homogenization PURE filter (Fast Spoiled Gradient Echo sequence, TR/TE/TI = 11.2/4.2/450 ms; flip angle 12°; 1 mm slice thickness, 256 × 256 matrix and FOV 25 cm).

### MEG acquisition and analysis

This section contains a summary of the MEG pipeline. A more detailed explanation of the acquisition, preprocessing, and source reconstruction procedure can be found in previous work [19]. Four minutes of resting state activity with eyes closed was recorded for each participant in a Vectorview MEG system (Elekta AB, Stockholm, Sweden) with 306 channels (102 magnetometers and 204 gradiometers) at the “Laboratory of Cognitive and Computational Neuroscience” (Madrid-Spain). Additionally, continuous head position and eye movements were also recorded. The acquired signal was filtered on-line with an anti-alias filter between 0.1 and 330 Hz and digitalized with a 1000 Hz sampling rate.

Recordings were offline filtered using spatiotemporal signal space separation algorithm (tSSS) with movement compensation using MaxFilter. Data was automatically inspected for artifacts, and the findings were manually confirmed by a MEG expert. When possible, artifact related to eye movements and heart activity were removed by ICA, and the related artifacts were removed accordingly. Artifact-free data were segmented into non-overlapping segments of 4 s. The analyses were conducted with magnetometers due to their high similarity with gradiometers after tSSS filter [25].

The source model consisted on a homogeneous grid of 1 cm defined in MNI space and labeled according to a compact version of the Automated Anatomical Atlas (see Additional file 1: Table S1 for the complete set of regions). This template-based source model was linearly transformed to the individual T1 image of each participant. To solve the direct problem, we employed a three-shell Boundary Element Method (BEM-3) with the surfaces (brain, skull, and scalp) extracted from the individual T1 image. The resulting lead field was used to generate a Linearly Constrained Minimum Variance beamformer for the broad band (2 to 45 Hz) activity.

Source power spectra estimates were calculated between 2 and 45 Hz for each 0.5-Hz frequency step using a discrete prolate spheroidal sequence (DPSS) as tapers with 0.5 Hz of frequency smoothing. The relative power was then calculated by normalizing power values with the overall power in the broad band range. Afterwards, relative power for each subject and source was obtained for the alpha band. Power data were finally averaged across each of the 38 regions of interest (ROIs) of the atlas.

### Classification and validation

To test the ability of MEG source power analysis to correctly classify SCD and HC participants, we used regularized logistic regression with the Least Absolute Shrinkage and Selection Operator (LASSO) [26] as implemented in the R 3.5.0 [27] and the *glmnet* package [28] including age, gender, and alpha power in 38 ROIs using the default criteria and leave-one-out cross-validation for determining the penalty factor that is chosen to minimize the expected prediction error. Unlike other variable selection techniques, LASSO regression constraints the sum of the absolute regression coefficients and sets the coefficients of variables that least contribute to the prediction to zero effectively leading to variable selection. We used the penalized coefficients to obtain the predicted probability of SCD status for receiver operating characteristic (ROC) curve analysis with the associated area under the curve (AUC) measure for discrimination. We defined as the optimal cut-off value the point on the AUC where the sum of sensitivity and specificity was maximized.

Initially, we performed the classification analysis on the whole sample to test the ability of alpha band activity obtained from MEG to correctly distinguish individual subjects of both groups. In a second step, we randomly split the study sample in a 2/3 discovery sample and 1/3 validation sample and repeated the above analysis to gain an insight into the external validity of the performance of MEG source results in the identification of SCD cases. Lastly, with the aim of proving the validity of our classifier do detect the early stages of cognitive decline in external samples, we employed the LASSO algorithm trained in the previous step (discovery-test sample) to test whether we could correctly distinguish a new sample of MCI participants from the healthy controls contained in the test sample. As an additional verification, we ensured that our classifier using alpha power was able to outperform a classifier merely based on neuropsychological scores differing at the group level between the control group and SCD.

## Results

In total, we obtained valid results for 161 participants (*n* = 70 HC, *n* = 91 SCD) (Table 1). SCD participants were on average 1.8 years older and more likely to be female. All SCD participants performed within the normal range in clinical assessment. As expected, SCD group scored quite significantly higher in the SCD-questionnaire (*p* = 0.00006). The 91 MCI participants used to validate the algorithm were on average 72.8 years old and 56 of them were female.

**Table 1:** Relevant demographic and clinical variables in the sample

	Mean ± SD			*p* values
	HC	SCD	MCI	HC vs SCD
Age	70.6 (4.4)	72.3 (5.2)	72.8 (4)	0.031
Gender (M-F)	29–41	20–71	35–56	0.008*
APOE (pos-neg)	15–49	16–65	36–51	0.59
GDS	1.4 (1.8)	3.0 (3.3)	3.7 (3.0)	0.001*
MMSE (gral. cognition)	28.9 (1.2)	28.2 (1.8)	26.9 (2.3)	0.104
RBMT global (episodic memory)	10.0 (1.6)	9.3 (2.4)	5.8 (3.2)	0.13
Direct digit (working memory)	8.5 (2)	8.4 (1.9)	7 (2.1)	0.660
Inverse digit (working memory)	6.2 (1.8)	5.2 (1.8)	4.2 (1.4)	0.009*
TMTA (hits) (executive funct.)	23.9 (0.3)	23.9 (0.5)	23.9 (0.9)	0.866
TMTB (hits) (executive funct.)	23.2 (2.5)	22.4 (3.1)	19.1 (6.1)	0.211
Gesture imitation (Praxis)	7.8 (0.6)	7.5 (0.9)	7.1 (1.3)	0.184
7 M-fluency (language)	20.5 (4.7)	18.6 (4.8)	13.7 (3.9)	0.140
7 M-clock (Praxis)	6.7 (0.5)	6.4 (1.1)	5.9 (1.4)	0.084

Age is compared using independent samples *t* test. Gender and APOE status (expressed as presence–absence of APOE4 allele) are compared using chi-squared. Neuropsychological test contrasts are adjusted by age and education. Each test is followed by the main cognitive domain intended to measure

*p < 0.05

**Fig. 1:**
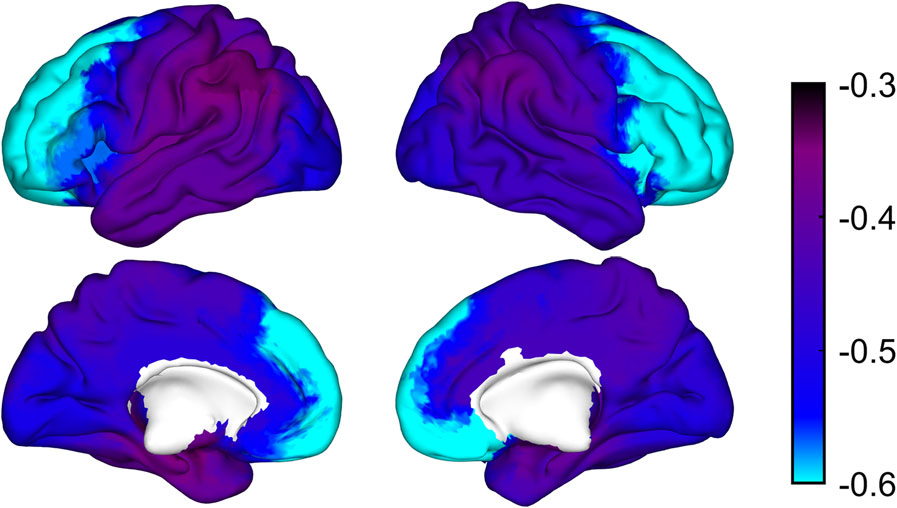
Figure displays the results of the age and gender adjusted regression of SCD status on each ROI independently. Color scale represents logarithmic odd ratios with negative values indicating an increase in SCD status risk associated to lower alpha power

**Fig. 2:**
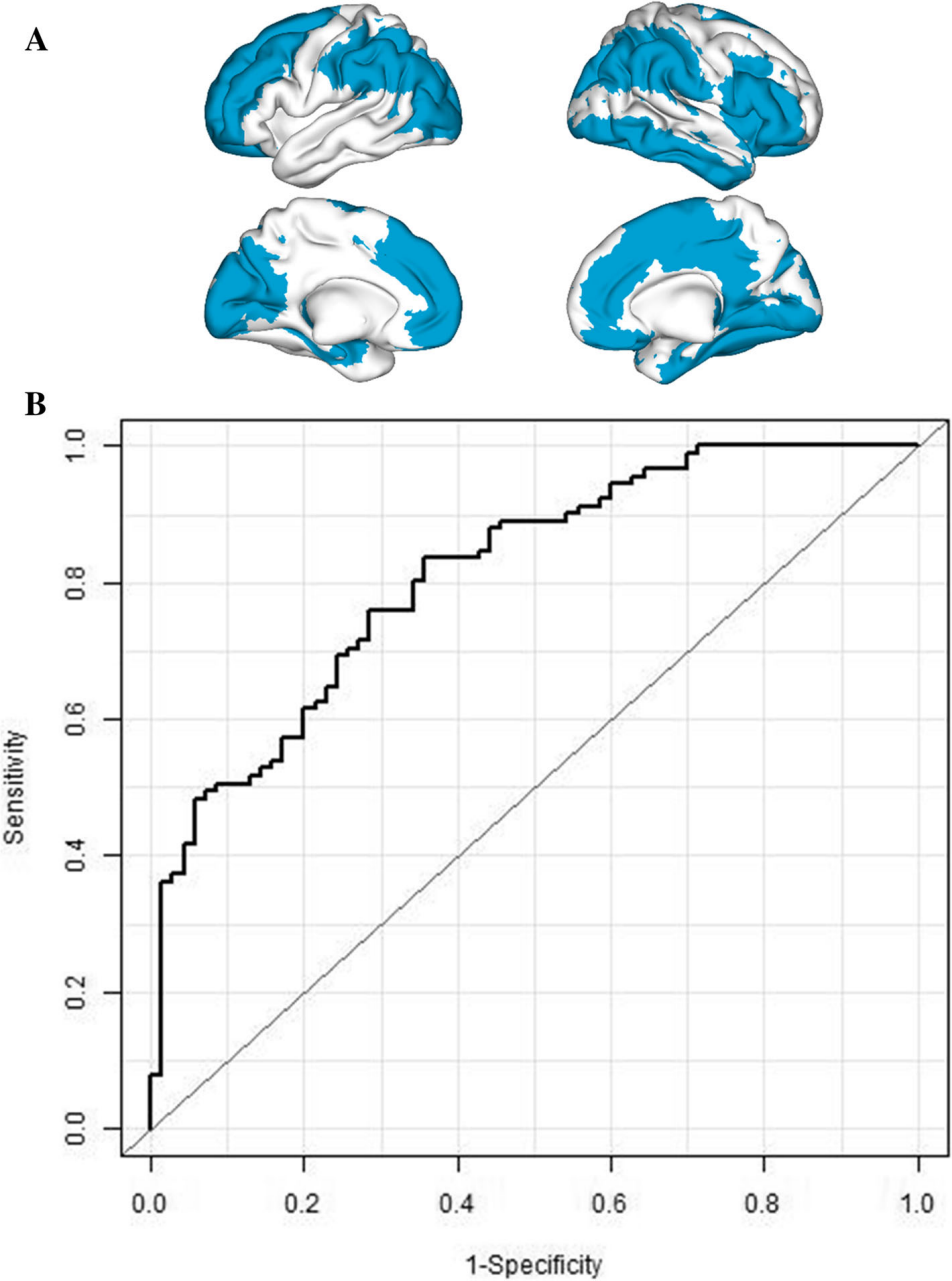
Figure displays the results for the whole sample discrimination analysis (SCD vs HC). **a** Top part shows the regions selected by the algorithm for classification. **b** Bottom part shows the ROC curve resulting of the LASSO algorithm

The age and gender adjusted results from the logistic regression of SCD status on each of the 38 ROIs separately are graphically displayed in Fig. 1 showing a generalized increase in SCD risk associated with lower alpha power values. LASSO regression selected age, gender, and the following ROIs as joint predictors of SCD status: left superior and middle frontal, right inferior frontal gyrus, right inferior temporal, left hippocampus, right superior and inferior occipital, left middle occipital, bilateral inferior parietal, right supplementary motor area, and right cingulum (see Fig. 2a and Additional file 2: Table S2 for the biased coefficients obtained from LASSO regression). The predicted probability of SCD status obtained from LASSO was significantly associated with SCD status (*p* < 0.001), and the AUC was 0.81 (95% CI 75.8–87.7%). Sensitivity and specificity at the point that maximized the sum of both were 83.5% (95% CI 75.8–91.2%) and 64.3% (95% CI 52.9–75.7%), respectively (Fig. 2b).

**Fig. 3:**
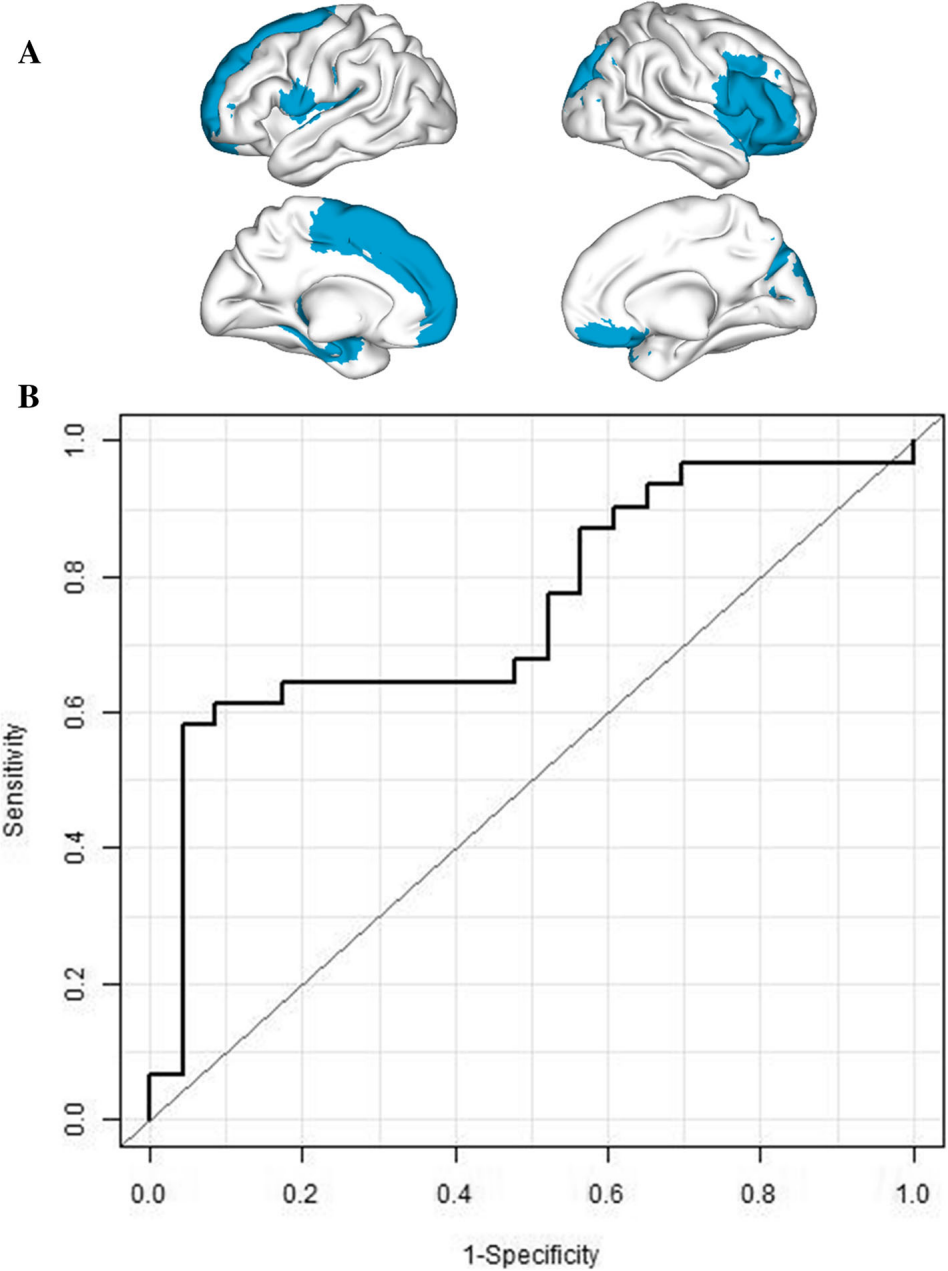
Figure displays the results for the discovery-test split samples discrimination analysis (SCD vs HC). **a** Top part shows the regions selected in the discovery sample by the algorithm for classification. **b** Bottom part shows the ROC curve resulting of the LASSO algorithm applied to the test sample

After randomly splitting the study sample into a training sample of *n* = 107 (60 SCD, 47 HC) and a validation sample of *n* = 54 (31 SCD, 23 HC) and repeating the LASSO analysis, the following ROIs were selected in the training sample for validation: left superior frontal, right inferior frontal, right superior occipital, left rolandic operculum, left supplementary motor area, and left hippocampus (Fig. 3a for a graphic representation and Additional file 3: Table S3 for the tabular format). When calculating the probability of SCD in the validation sample by applying the LASSO results from the training sample, the classifier was significantly associated to SCD status in the validation sample (*p* = 0.005) with an associated AUC of 75.3% (95% CI 61.9–88.7%). Sensitivity and specificity at the point that maximized the sum of both were 58.9% (95% CI 41.9–74.2%) and 95.7% (95% CI 87.0, 1.00) respectively (Fig. 3b). This model was significantly different from using age and gender alone for prediction (AUC = 60.1%, *p* = 0.156), p_DeLong_ for difference = 0.016) and from using age, gender, and working memory (AUC = 61.4%, *p* = 0.125), p_DeLong_ for difference = 0.049). This result shows that alpha power is able to outperform the accuracy of neuropsychological testing and sociodemographic variables in distinguishing older adults with SCD from healthy controls.

To confirm the validity of the derived predictor, we applied calculated probability of having SCD status obtained from the training sample as a predictor to discriminate between 23 HC from the validation sample and 91 independent participants with MCI. The SCD predictor was significantly associated to MCI status (*p* = 0.03), sensitivity (36.3%, 95% CI 26.3, 46.2%) was low, but specificity (95.7%, 95% CI 87.0–100%) was high meaning that participants classified as not having MCI were very likely true negatives at the point that maximized the sum of both.

## Discussion

The current study demonstrated that magnetoencephalography, and more concretely the power spectral properties of ongoing brain activity, can be a useful tool for distinguishing individuals with SCD and healthy older adults without cognitive concerns. To the best of our knowledge, this is the first time that electrophysiological brain activity is used to characterize SCD on an individual basis. Our results using the algorithm on the whole sample showed great accuracy (over 80%). More importantly, when the sample was split, the newly obtained classifier from the reduced training sample was able to discriminate SCD individuals with more than 75% accuracy in the independent validation sample, thus showing good external validity of relative alpha power as a potential candidate to identify very early synaptic dysfunction. Importantly, although with a lower accuracy but high specificity, the SCD algorithm was also significantly associated to MCI, a well-known at-risk state for dementia. Given that SCD and MCI have been linked to an increased risk of dementia and cognitive decline, the significant association of our classifier with both populations offers additional evidence of its relevance in the context of early cognitive dysfunction, and potentially for the preclinical stages of AD. Furthermore, this work expands previous findings into the individual level, a crucial step for biomarker detection.

Previous neuroimaging studies have already identified alterations in older adults with SCD, resembling those abnormalities found in later stages of dementia due to AD, such as increased levels of amyloid and tau proteins [29], cortical atrophy [30], or functional connectivity alterations [31]. These studies support our results reporting a relationship between the alterations found in SCD and MCI populations compared to healthy older adults and pinpoint the possibility of both states being part of a continuum towards dementia. Electrophysiological research, in particular, has identified a shift to the left in the power spectrum in AD patients that correlates with disease severity [32]. These alterations are characterized by a power increase in slower rhythms and a decrease in faster waves, such as alpha oscillations. Interestingly, most of the regions selected by LASSO classifier such as bilateral frontal and ventromedial frontal, inferior parietal regions, and the cingulate gyrus, or more concretely its posterior aspect, are typically known to exhibit cortical hypo-metabolism as reflected by FDG-PET specifically associated to AD [33]. Furthermore, frontal regions seem to play a crucial role in the amyloid network even in the very early stages of AD [34]. Consistent with our interpretation of SCD as an at-risk state potentially leading to dementia, a recent study by Babiloini et al. [35] also identified a decrease in alpha power over these same cortical regions in AD patients. Moreover, a review conducted by the same group has recently proposed alpha power alterations as a potential good candidate for the detection of AD or its prodromal phases [36], which seems to be supported in our results.

Alpha oscillations during awake rest are known to govern a wide range of relevant functions for cognition, from general brain arousal and global attentional readiness to particular cognitive functions such as memory or sensorimotor networks [36]. In fact, disturbances in alpha oscillations reflect synaptic dysfunction, which is thought to be the best correlate of early cognitive impairment in AD [37]. In this vein, the alpha relative power reduction observed in our sample of SCD could represent an early indicator of very subtle synaptic alteration that is not yet manifested as an objective cognitive deficit at this stage. Furthermore, a recent study showed that when stratified for diagnosis, alpha power reduction measured with EEG was able to predict future cognitive decline and conversion in healthy older adults with SCD [38]. Although longitudinal studies are necessary to confirm this hypothesis, those subjects selected by our algorithm may represent a specific subpopulation among older adults with SCD at a highly increased risk of subsequent cognitive decline.

Abnormal AD-related protein accumulation in the brain of preclinical and fully demented AD patients has been shown to produce alterations in power spectrum [37]. In this study, alpha power reductions were associated to decreased Aβ levels, and increased total and phosphorylated tau levels in cerebrospinal fluid (CSF) already in the SCD and MCI stages. However, results in this regard are yet scarce and a recent study reported alpha power increases in relation to Aβ accumulation over frontal regions [18], although in this same study MCI participants exhibited a significant reduction of relative alpha power which is in agreement with our findings. In accordance with the former study, and despite the limitation that no CSF biomarkers were available in our sample, the power spectral alterations found in our sample might be an early sign of the initial neuropathology accumulation, leading to a decrease in alpha power. This would be in line with the fact that SCD participants are known to show increased levels of tau and Aβ burden as shown by Buckley et al. [39].

Very little literature is available addressing SCD individual classification, and to the best of our knowledge, none of them employed electrophysiological data, but structural and functional MRI information instead. One of them obtained a significant classification with 0.96 AUC [40] using entorhinal volume and fractional anisotropy of the hippocampal body. However, its small sample size and the fact that there was no test sample (i.e., feature selection and test was performed in the same sample) limits its reliability. A previous study employed an algorithm trained with MRI data from AD patients, to then distinguish healthy elders from SCD [16]. They obtained a significant classifier with 0.67 AUC, which is a similar value to what we obtained when we employed our SCD-HC classifier in the MCI sample. The hippocampus was the region with higher contribution in the above-mentioned classification, which was again replicated in a posterior study [41]. Interestingly, our results also highlight hippocampus, a central structure in AD neurodegeneration [42], as one of the key regions in distinguishing between both populations. This is consistent with previous work reporting structural decline with alpha power reduction [43]. A recent study was also able to significantly classify SCD participants from their BOLD signals, although completely independent samples were again not included for validation [44].

This study is not absent from certain limitations. First, despite its relatively large sample size within the field of MEG literature, the number of participants is still somewhat limited for a study of individual classification. Furthermore, although split in a discovery and a validation sample, both belong to the same study population, which could potentially limit its replicability and highlights the need of validation in different population settings. However, fully detailed outcomes of the algorithm are available on request in order to replicate our findings in new samples and different acquisition settings. Lastly, it is important to bear in mind that the absence of AD-biomarkers hinders our ability to unequivocally associate our findings to the presence of this pathology. Thus, in spite of the careful sample selection (following up-to-date established criteria and ruling our variables such as depression or other neurological conditions), we cannot completely dismiss the possibility that other conditions may influence our results. In this regard, future studies should further validate the present findings with additional biomarker evidence.

## Conclusions

In conclusion, our results suggest that MEG and electrophysiology represent promising tools for the early detection of pathological aging and in particular AD. This could be of crucial interest for policymakers and future pharmacological studies, as MEG could represent a potential good candidate to select a targeted subset of the population in which treatments or preventive interventions resulted potentially more beneficial. Furthermore, this MEG-informed signature could potentially be translatable to EEG which would result in a cheaper and more broadly available risk assessment tool.

## Additional files

Additional file 1: **Table S1.** List of ROIs. (DOCX 14 kb)

Additional file 2: **Table S2.** LASSO results for the whole sample. (DOCX 14 kb)

Additional file 3: **Table S3.** LASSO results for the discovery sample. (DOCX 14 kb)
