# Supplementary material for: Electrophysiological brain signatures for the classification of subjective cognitive decline: towards an individual detection in the preclinical stages of dementia
Source: Alzheimers Res Ther. 2019 Jun 1;11:49. doi: 10.1186/s13195-019-0502-3 (PMC6544924; doi:10.1186/s13195-019-0502-3)
Supplement: Supplementary file 1 — Table S1. List of ROIs. (DOCX 14 kb) [file 13195_2019_502_MOESM1_ESM.docx]

Additional file 1: **Table S1**. List of ROIs

| **List of ROIs** |
| --- |
| Left superior frontal |
| Right superior frontal |
| Left middle frontal |
| Right middle frontal |
| Left inferior frontal |
| Right inferior frontal |
| Left superior temporal |
| Right superior temporal gyrus |
| Left middle temporal |
| Right middle temporal |
| Left inferior temporal |
| Right inferior temporal |
| Left superior occipital |
| Right superior occipital |
| Left middle occipital |
| Right middle occipital |
| Left inferior occipital |
| Right inferior occipital |
| Left precuneus |
| Right precuneus |
| Left superior parietal |
| Right superior parietal |
| Left inferior parietal |
| Right inferior parietal |
| Left precentral |
| Right precentral |
| Left postcentral |
| Right postcentral |
| Left rolandic operculum |
| Right rolandic operculum |
| Left supplementary motor |
| Right supplementary motor |
| Left cingulum |
| Right cingulum |
| Left hippocampus |
| Right hippocampus |
| Left parahippocampal |
| Right parahippocampal |

Supplementary Table S1.

Complete list of ROIs included in the analysis
