# Supplementary material for: Electrophysiological brain signatures for the classification of subjective cognitive decline: towards an individual detection in the preclinical stages of dementia
Source: Alzheimers Res Ther. 2019 Jun 1;11:49. doi: 10.1186/s13195-019-0502-3 (PMC6544924; doi:10.1186/s13195-019-0502-3)
Supplement: Supplementary file 2 — Table S2. LASSO results for the whole sample. (DOCX 14 kb) [file 13195_2019_502_MOESM2_ESM.docx]

Additional file 2: **Table S2.** LASSO results for the whole sample

| **Variable** | **Log(OR)** |
| --- | --- |
| Intercept | 0.320 |
| Age | 0.460 |
| Gender | 0.719 |
| L. Sup. Frontal | -0.610 |
| L. Mid. Frontal | -0.313 |
| R. Inf. Frontal | -1.022 |
| R. Inf. Temporal | -0.122 |
| R. Sup. Occipital | -0.294 |
| L. Mid. Occipital | -0.405 |
| R. Inf. Occipital | -0.083 |
| L. Inf. Parietal | 0.976 |
| R. Inf. Parietal | 0.066 |
| R. Supp. Motor | -0.466 |
| R. Cingulum | 0.570 |
| L. Hippocampus | 1.147 |

Supplementary Table S2

List of variables selected by LASSO algorithm to distinguish SCD and HC in the discovery sample and applied to the test sample. L and R stands for left and right respectively.
