# Supplementary material for: Electrophysiological brain signatures for the classification of subjective cognitive decline: towards an individual detection in the preclinical stages of dementia
Source: Alzheimers Res Ther. 2019 Jun 1;11:49. doi: 10.1186/s13195-019-0502-3 (PMC6544924; doi:10.1186/s13195-019-0502-3)
Supplement: Supplementary file 3 — Table S3. LASSO results for the discovery sample. (DOCX 14 kb) [file 13195_2019_502_MOESM3_ESM.docx]

Additional file 3: **Table S3.** LASSO results for the discovery sample.

| **Variable** | **Log(OR)** |
| --- | --- |
| Intercept | -8.352 |
| Age | 0.092 |
| Gender | 1.232 |
| L. Sup. Frontal | -0.259 |
| R. Inf. Frontal | -0.646 |
| R. Sup. Occipital | -0.238 |
| L. Rolandic Operculum | 0.361 |
| L. Supp. Motor | -0.018 |
| L. Hippocampus | 0.409 |

Supplementary Table S3

List of variables selected by LASSO algorithm to distinguish SCD and HC in the whole sample analysis. L and R stands for left and right respectively.
